# Whole-genome-sequence-based characterization of an NDM-5-producing uropathogenic *Escherichia coli* EC1390

**DOI:** 10.1186/s12866-022-02562-6

**Published:** 2022-06-06

**Authors:** Tran Thi Dieu Thuy, Hsu-Feng Lu, Pei-Yun Kuo, Wei-Hung Lin, Tzu-Ping Lin, Yi-Tzu Lee, Tran Thi Thuy Duong, Ming-Cheng Wang, Yi-Hong Lee, Li-Li Wen, Yu-Chen Chen, Cheng-Yen Kao

**Affiliations:** 1 Institute of Microbiology and Immunology, College of Life Sciences, National Yang Ming Chiao Tung University, No.155, Sec.2, Linong Street, Taipei, 112 Taiwan; 2 Department of Laboratory Medicine, China Medical University Hospital, Taichung, Taiwan; 3 Department of Medical Laboratory Science and Biotechnology, Asia University, Taichung City, Taiwan; 4 Department of Internal Medicine, College of Medicine, National Cheng Kung University Hospital, National Cheng Kung University, Tainan, Taiwan; 5 Department of Urology, School of Medicine, National Yang Ming Chiao Tung University, Taipei, Taiwan; 6 Department of Emergency Medicine, Taipei Veterans General Hospital, Taipei, Taiwan; 7 Department of Clinical Laboratory, En Chu Kong Hospital, New Taipei City, Taiwan; 8 Genomics Research Center, Academia Sinica, Taipei, Taiwan

**Keywords:** *bla*_NDM-5_, Extensively-drug resistant, Urinary tract infection, Virulence, Whole-genome sequencing

## Abstract

**Background:**

Urinary tract infection (UTI) is one of the most common outpatient bacterial infections. In this study, we isolated and characterized an extensively-drug resistant (XDR) NDM-5-producing *Escherichia coli* EC1390 from a UTI patient by using whole-genome sequencing (WGS) in combination with phenotypic assays.

**Methods:**

Antimicrobial susceptibility to 23 drugs was determined by disk diffusion method. The genome sequence of EC1390 was determined by Nanopore MinION MK1C platform. Conjugation assays were performed to test the transferability of EC1390 plasmids to *E. coli* recipient C600. Phenotypic assays, including growth curve, biofilm formation, iron acquisition ability, and cell adhesion, were performed to characterize the function of EC1390 plasmids.

**Results:**

Our results showed that EC1390 was only susceptible to tigecycline and colistin, and thus was classified as XDR *E. coli*. A de novo genome assembly was generated using Nanopore 73,050 reads with an *N*_50_ value of 20,936 bp and an *N*_90_ value of 7,624 bp. WGS analysis showed that EC1390 belonged to the O101-H10 serotype and phylogenetic group A *E. coli*. Moreover, EC1390 contained 2 conjugative plasmids with a replicon IncFIA (pEC1390-1 with 156,286 bp) and IncFII (pEC1390-2 with 71,840 bp), respectively. No significant difference was observed in the bacterial growth rate in LB broth and iron acquisition ability between C600, C600 containing pEC1390-1, C600 containing pEC1390-2, and C600 containing pEC1390-1 and pEC1390-2. However, the bacterial growth rate in nutrition-limited M9 broth was increased in C600 containing pEC1390-2, and the cell adhesion ability was increased in C600 containing both pEC1390-1 and pEC1390-2. Moreover, these plasmids modulated the biofilm formation under different conditions.

**Conclusions:**

In summary, we characterized the genome of XDR-*E*. *coli* EC1390 and identified two plasmids contributing to the antimicrobial resistance, growth of bacteria in a nutrition-limited medium, biofilm formation, and cell adhesion.

**Supplementary Information:**

The online version contains supplementary material available at 10.1186/s12866-022-02562-6.

## Background

Urinary tract infection (UTI) is one of the most common outpatient bacterial infection, with a lifetime incidence of 50–60% in adult women. Therefore, UTI causes a substantial financial burden on society [1]. *Escherichia coli*, *Klebsiella pneumoniae*, *Proteus mirabilis*, *Enterococcus faecalis*, and *Staphylococcus saprophyticus*, are common pathogens isolated from UTI patients [2]. Among these pathogens, uropathogenic *E. coli* (UPEC) is the dominant infectious pathogen in both uncomplicated and complicated UTIs [2]. The great diversity in gene content, genomic organization, pathogenicity islands, and virulence factors of UPEC strains are associated with their uropathogenesis [3]. Carriage of urovirulence factors such as K1 capsule, adhesins PapGI-III, Afa, FimH fimbriae, cytotoxic necrotizing factor (Cnf1), hemolysin, uropathogenic specific protein (Usp), and outer membrane protease T (OmpT) are thought to enhance UPEC virulence and are used to measure and categorize clinical UPEC strains [4, 5].

Cephalosporins, fosfomycin, trimethoprim, fluoroquinolones, and amoxicillin in combination with β-lactamase inhibitors, are considered effective antibiotics to reduce the duration of *E. coli*-causing UTI symptoms in the last decade [6]. However, the extensive use of antibiotics has led to the emergence of antibiotic-resistant UTI pathogens [7]. *E. coli* isolated from patients with UTIs showed relatively low resistance to carbapenems (imipenem, ertapenem, meropenem, and doripenem), therefore, carbapenems were considered the drug of last resort for the treatment of UTIs caused by extended-spectrum β-lactamases-producing *E. coli* [8]. The mechanisms of carbapenem resistance in *Enterobacteriaceae* are strongly associated with carbapenemase production, overexpression of efflux pumps, and porin loss [9]. Moreover, the emergence of conjugative plasmids carrying carbapenemase has been increasingly reported among *Enterobacteriaceae* and is a matter of major clinical concern [10]. Currently, the application of next-generation sequencing (NGS) extends from microbial identification to epidemiological insight, microbial community investigation, and antimicrobial resistance prediction [11]. Therefore, in this study, we performed whole-genome sequencing (WGS) in combination with phenotypic assays to characterize an NDM-5-producing *E. coli* strain EC1390 isolated from a UTI patient.

## Methods

### Sampling and isolation of *E. coli*

*E. coli* isolates were recovered from patients with UTIs at National Cheng Kung University Hospital (NCKUH) with the approval of the NCKUH Research Ethics Committee (Approval No: B-ER-110–144). Informed consent was waived because of the retrospective nature of the study, the isolates were remnants from patient samples, and the analysis used anonymous clinical data. A total of 844 non-duplicate *E. coli* isolates were identified in the clinical laboratory by colony morphology, Gram stain, biochemical tests, and the Vitek system (bioMérieux, Marcy l'Etoile, France) according to the manufacturer's recommendations [12]. *E. coli* isolates were stored at − 80 °C in lysogeny broth (LB) containing 20% glycerol (v/v) until tested.

### Antimicrobial susceptibility testing

The antimicrobial susceptibility was determined by disk diffusion assay against 23 antibiotics ((amikacin (30 μg), amoxicillin (30 μg), ampicillin (10 μg), ampicillin/sulbactam, cefazolin (30 μg), cefepime (30 μg), cefixime (5 μg), cefmetazole (30 μg), cefoperazone/sulbactam (10/10 μg), cefoxitin (30 μg), ceftazidime (30 μg), ceftriaxone (30 μg), cefuroxime (30 μg), ciprofloxacin (5 μg), ertapenem (10 μg), gentamicin (10 μg), imipenem (10 μg), meropenem (10 μg), levofloxacin (5 μg), piperacillin/tazobactam (100/10 μg), sulfamethoxazole/trimethoprim (23.75/1.25 μg), tetracycline (30 μg), and tigecycline (15 μg)) (BD BBL Sensi-Disc, Becton, Dickinson and Company, MD, U.S.A.) following the Clinical and Laboratory Standards Institute (CLSI) guidelines [13]. The Vitec 2 system (Biomérieux) was used to determine the minimal inhibitory concentrations (MICs) of EC1390 and transconjugants (TCGs) to antibiotics according to the manufacturer's instructions. *E. coli* ATCC 25922 was used as a quality control strain. The interpretation of resistance to these antimicrobial agents was determined according to the recommendations of the CLSI [13].

### Genome sequencing, assembly, annotation, and analysis

The genomic DNA (gDNA) of EC1390 was extracted using a Presto™ gDNA Bacteria Advanced Kit (Geneaid Biotech, Ltd., Taiwan) from a 5 mL broth culture grown in LB, followed by the protocol for Gram-negative bacteria. One μg gDNA was used to construct the sequencing library by using a Ligation Sequencing Kit (SQK-LSK109, Oxford Nanopore techonologies, Oxford, UK). KAPA HyperPure beads purchased from Oxford Nanopore techonologies were used to purify the gDNA fragments. The Oxford Nanopore MinION MK1C was used to determine the whole genome sequence of EC1390. A total of 300 ng gDNA was loaded onto R9.4.1 flow cell. The quality of reads generated was assessed using Fast QC v0.11.5 (https://www.bioinformatics.babraham.ac.uk/projects/fastqc/). Raw signals were translated into a DNA sequence by using ONT Gussy basecalling program (version 4.2.3). EC1390 genome was constructed with Flye de novo assembler (version 2.9), and the options for backward compatibility (plasmids) and uneven coverage mode (meta) were used in this analysis [14].

Genome annotation was performed by NCBI Prokaryotic Genome Annotation Pipeline (PGAP) (https://www.ncbi.nlm.nih.gov/genome/annotation_prok/). CGView Server was used to generate graphical maps of circular genomes and plasmids (http://stothard.afns.ualberta.ca/cgview_server/). Rapid Annotations using Subsystems Technology (RAST, https://rast.nmpdr.org/rast.cgi) was used to predict the subsystem of genes in EC1390 genome (annotation scheme, classic RAST; gene caller, RAST; FIGfam version, Release70). The multilocus sequence typing (MLST), ResFinder, and PlasmidFinder databases (http://www.genomicepidemiology.org/) were used to find the sequence type (ST), serotype, antibiotic resistance genes, and the plasmid types present in EC1390. Insertion sequences (ISs) and CRISPR-Cas were characterized using the IS Finder database (https://www-is.biotoul.fr/) (data base, ISfinder; programme, blastn) and CRISPR Finder database (https://crispr.i2bc.paris-saclay.fr/Server/), respectively. CRISPRTarget was used to identify the target of spacers (http://crispr.otago.ac.nz/CRISPRTarget/crispr_analysis.html).

### Phylogenetic analysis of pEC1390-1 and pEC1390-2 closely related plasmids

The sequences of assembled plasmids, pEC1390-1 and pEC1390-2, were submitted to BLAST and compared to previously sequenced plasmids in NCBI GenBank database. Top twenty-five plasmids with the highest degree of similarity to the plasmid sequences of pEC1390-1 and pEC1390-2 (also with greater than 70% query coverage), respectively, were selected to perform CulstalW alignment (Gap opening penalty, 15.00; Gap extension penalty, 6.66) to construct Maximum Likelihood trees inferred with the Jukes-Cantor model by using Molecular Evolutionary Genetics Analysis (MEGA) software version 11.0. Bootstrap confidence values were obtained applying 300 replications.

### Conjugation experiments and plasmid analysis

The liquid mating-out assay was used to determine the conjugation ability of plasmids pEC1390-1 and pEC1390-2, to rifampin-resistant *E. coli* C600 as described previously [15]. TCGs were selected on LB plates containing rifampin (256 μg/mL) combined with ampicillin (100 μg/mL) or chloramphenicol (25 μg/mL). Plasmid numbers in the parental strains and TCGs were determined by using Kado-Liu extraction methods as described previously [16]. *Salmonella* OU7526 and *E. coli* EC974/EC1515 contained 2 (50 and 90 kbp) and 3 (78, 92, and 105 kbp) plasmids, respectively, were used as plasmid size controls [15, 17]. PCR with *bla*_NDM_ primers was used to detect the presence of *bla*_NDM_ in the TCGs [18].

### Phenotypic detection of carbapenemase

mCIM and eCIM were performed on EC1390 and TCGs to detect the presence of carbapenemase [19]. A 1-μL loopful of bacteria was resuspended in a 2-mL tube of tryptone soy broth (TSB) (BD Difco, Detroit, MI, USA). Another 1-μL loopful of bacteria was resuspended in a 2-mL tube of TSB supplemented with EDTA (Thermo Fisher Scientific, Carlsbad, CA, USA) at a final concentration of 5 mM. A meropenem disk was placed in each tube, and the tubes were incubated at 35 °C for 4 h ± 15 min. Subsequently, the disks were removed and applied to Mueller–HInton agar plates (BD Difco) freshly plated with a 0.5 McFarland suspension of a carbapenem-susceptible *E. coli* ATCC 25922 strain. The plates were incubated at 35 °C for 16 to 20 h and the mCIM and eCIM results were interpreted as previously described [19]. *K. pneumoniae* ATCC BAA-1706 (carbapenemase-negative), *K. pneumoniae* ATCC BAA-1705 (*bla*_KPC_-positive), and *K. pneumoniae* ATCC BAA-2146 (*bla*_NDM_-positive) were used as internal controls for mCIM and eCIM tests.

### Growth of bacteria in M9 and LB medium

The rate of growth was determined over 24 h at 37 °C using a BioTek Synergy HTX multimode reader [20]. 200 μL of sterile M9 (BD Difco) or LB (Neogen, Lansing, Michigan, USA) broth was dispensed into clear sterile 96-well microplates and inoculated with overnight culture of each strain to give a final inoculum of 1%. Readings were taken every fifteen minutes of absorbance of each well (scanned at OD_600_ nm) in the microplates over the 24 h period. Each strain was analyzed in triplicate wells on at least three separate occasions to give nine data sets for analysis.

### Biofilm formation assay

Biofilm formation by *E. coli* was assayed using a 96-well flat-bottom polystyrene microtiter plate, as described previously [21], with some modifications. Biofilms were stained with 0.1% crystal violet for 30 min. Crystal violet dye associated with biofilms was eluted with 75% ethanol for 30 min, and was quantified by absorbance at 590 nm.

### CAS agar diffusion assay

Chrome azurol S (CAS) agar plates were prepared as described previously [22]. The modified CAS agar plate was punched with 6.5-mm diameter holes by using a tip (Corning® Pasteur pipettes, no. L, 5 3/4 inch (146 mm)). Each hole was filled with 50 μL of the LB broth containing equal CFU of *E. coli*, incubated at 37˚C for 16 h, and monitored for the formation of an orange halo. CAS remains blue when complexed with iron, but it turns orange when iron is chelated by other iron chelators. Siderophore activity was expressed as the halo diameter.

### Cell line and adhesion assay

The bladder epithelial cell line 5637 was a kind gift of Prof. Ching-Hao Teng (National Cheng-Kung University, Taiwan). 5637 cells were seeded into 12-well plates in RPMI-1640 media (Thermo Fisher Scientific) with 10% fetal bovine serum (FBS) (HyClone Laboratories, Logan, UT, USA) and 1% penicillin–streptomycin (HyClone) and grown to a monolayer. Before 2 h of infection, the culture medium was replaced with fresh medium without 1% penicillin–streptomycin. The cells were infected with a MOI of 10 of the *E. coli* strains, centrifuged at 600 g for 5 min to synchronize bacteria-host cell contact, and incubated at 37 °C for 2 h. The cells were washed three times with phosphate buffered saline (PBS). The cells were lysed by incubation with saponin at 37 °C for 15 min and plated on LB plates. The resulting colonies were counted to determine the adherent bacteria [23].

### Statistical analysis

All analyses were performed in GraphPad Prism 8.0 software (GraphPad Software, Inc., San Diego, CA). Student’s *t* test was performed to evaluate the statistical differences in phenotypic assay experiments. A value of *p* < 0.05 was considered statistically significant.

## Results

### Isolation of extensively-drug resistant EC1390

Among 844 UTI isolates, our antimicrobial susceptibility tests showed that 11 (1.3%), 221 (26.2%), 640 (75.8%), 265 (31.4%), 244 (28.9%), 30 (3.6%), 7 (0.8%), 9 (1.1%), 4 (0.5%), 259 (30.7%), 249 (29.5%), 99 (11.7%), 249 (29.5%), 199 (23.6%), 121 (14.3%), 154 (18.2%), 359 (42.5%), 286 (33.9%), 462 (54.7%), and 408 (48.3%) isolates were non-susceptible to amikacin, gentamicin, ampicillin, amoxicillin, ampicillin/sulbactam, piperacillin/tazobactam, imipenem, ertapenem, meropenem, cefazolin, cefuroxime, cefmetazole, ceftriaxone, ceftazidime, cefepime, cefoxitin, ciprofloxacin, levofloxacin, tetracycline, and sulfamethoxazole/trimethoprim, respectively. All 844 isolates were susceptible to tigecycline and colistin. Moreover, only one *E. coli* strain, EC1390, was resistant to three carbapenems, including imipenem, ertapenem, and meropenem. Moreover, EC1390 showed resistance to ampicillin, amoxicillin, amikacin, ceftazidime, ciprofloxacin, cefmetazole, ceftriaxone, cefuroxime, cefazolin, cefepime, cefoxitin, gentamicin, levofloxacin, ampicillin/sulbactam, sulfamethoxazole/trimethoprim, tetracycline, and piperacillin/tazobactam. EC1390 was only susceptible to tigecycline and colistin, therefore, EC1390 was classified as extensively-drug resistant (XDR) *E. coli*.

**Fig. 1:**
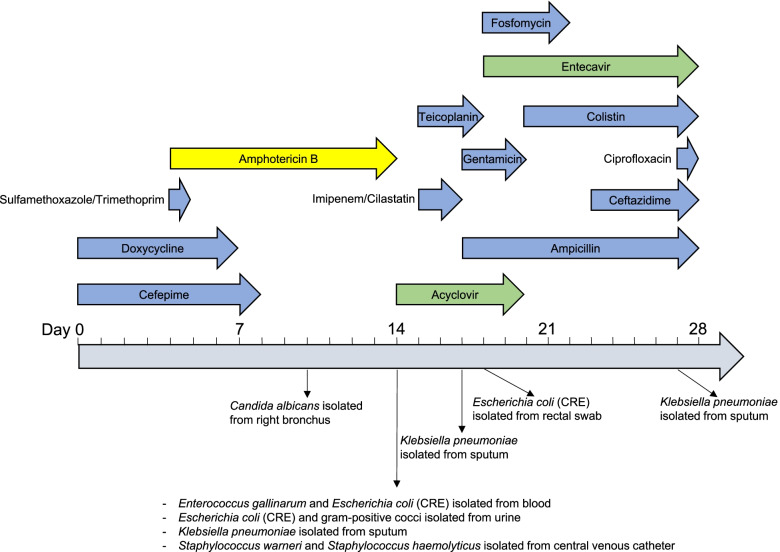
Timeline of the patient’s clinical treatment and pathogen isolation. EC1390 was isolated from the urine sample on day 14 of patient’s hospitalization. Antibiotics, antifungal drug, and antiviral drugs, are shown in blue, yellow, and green arrows, respectively

EC1390 was isolated from a 50-year-old woman admitting to NCKUH on September 4, 2020, for acute hypoxemic respiratory failure with acute respiratory distress syndrome. The patient also had chronic lymphocytic leukemia. The patient was hospitalized at NCKUH from September 4 to December 3. The timeline of the first 4 weeks of history of drug use and pathogen isolation of this patient was described in Fig. 1. EC1390 was isolated from the urine sample on day 14 of patient’s hospitalization. Cefepime (day 1–8), doxycycline (day 1–7), and sulfamethoxazole/trimethoprim (day 4), were used for the treatment of bacterial infections before EC1390 was isolated (Fig. 1).

### Complete genome sequence of EC1390 and its characteristics

**Fig. 2:**
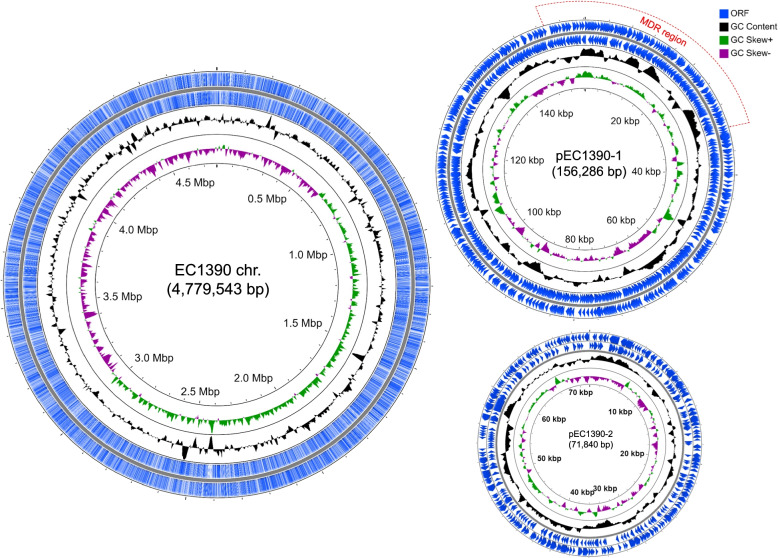
Circular genome map of *E. coli* EC1390. The scales indicate the location in Mbp (chromosome) or Kbp (plasmid), starting with the initial coding region. From the innermost circles, circle (1) shows the GC skew (G-C/G + C). The value is plotted as the deviation from the average GC skew of the entire sequence. Circle (2) shows the GC content, plotted using a sliding window. Circle (3, 4) illustrate the coding sequences, 3 is backward strand, 4 is forward strand. The region containing multiple drug resistance genes on pEC1390-1 was indicated

Nanopore WGS data to construct the EC1390 genome were obtained using the MinION flow cell according to the manufacturer’s protocol. A de novo assembly was generated using Nanopore 73,050 reads with an *N*_50_ value of 20,936 bp and an *N*_90_ value of 7,624 bp (mean coverage was 316). Reads were assembled and returned three contigs with the head segment was almost identical to the tail segment, indicating the circular nature of the contigs. The closed EC1390 genome comprises a 4,779,543-bp chromosome, a 156,286-bp plasmid named pEC1390-1, and a 71,840-bp plasmid named pEC1390-2 (Fig. 2 and Table 1). The number of plasmid in EC1390 was verified by Kado-Liu's plasmid extraction method (Fig. S1), however, 3 putative plasmids with an expected size lower than 10 kbp were not identified by Nanopore WGS. GC content of the EC1390 chromosome was 50.8% and that of plasmid pEC1390-1 and pEC1390-2 was 52.3% and 51.9%, respectively (Table 1).

**Table 1:** Characteristics of chromosome and plasmids in EC1390

Chromosome	Size (bp)	GC contents (%)	ORF (n)	RNA (n)	
EC1390	4,779,543	50.8	5,002	118	
Plasmids	Size (bp)	GC contents (%)	ORF (n)	Replicon	Conjugative related genes
pEC1390-1	156,286	52.3	192	IncFIA	+
pEC1390-2	71,840	51.9	94	IncFII	+

NCBI PGAP annotation results showed the EC1390 chromosome contained 5,002 coding sequences and 118 RNA genes (Table 1). 192 and 94 protein-coding sequences were identified on the plasmids pEC1390-1 and pEC1390-2, respectively (Table 1). However, RAST annotation results showed that the EC1390 chromosome contained 10,738 coding sequences and 110 RNA genes. Moreover, 6,821 protein-coding sequences (64%) were functionally annotated by RAST server in 599 subsystems (Fig. S2A). 129 protein-coding sequences (33%) of pEC1390-1 were functionally annotated by RAST server in 13 subsystems (Fig. S2B) while the protein-coding sequences of EC1390-2 could not be incorporated into any subsystems.

Serotype was determined by the genotype of *fliC, wzx*, and *wzy*, and the results showed that EC1390 belonged to the O101-H10 serotype. Phylogenetic group analysis showed that EC1390 was group A *E. coli*. Acquired antimicrobial resistance genes were not identified on EC1390 chromosome sequence. However, an amino acid substitution E84K of ParC associated with nalidixic acid and quinolone resistance was identified on EC1390 chromosome sequence. The MLST scheme of EC1390 which is based on the sequences of seven housekeeping genes (*gyrB999, recA2, mdh8, purA13, icd996, adk922, fumC11*) confirmed that EC1390 belonged to a novel sequence type, and the results were further verified by sanger’s sequencing.

To characterize CRISPR-Cas systems in EC1390, the presence of true CRISPRs in the EC1390 genome was assessed by the CRISPRCasFinder tool. To discriminate spurious CRISPR-like elements from the true CRISPRs, only CRISPRs classified with evidence levels 4 were considered for further analyses. Based on the selection criteria, one CRISPR type IE loci/arrays containing 10 spacers were detected in EC1390 (Table S1). To look for potential targets of EC1390 CRISPR spacers, CRISPRTarget was used to find the spacer against the RefSeq-Plasmid and PHAST databases. Three spacer sequences (TGTGTTTGCGGCATTAACGCTCACCAGTATTTC, CGACGTGGTCATGGGTGCTGCTGTTGCAGAGCCA, and CGAATCGCGCATACCCTGCGCGTCGCCGCCTGC) were shown to target plasmids and/or phages (Table S1). However, the function/activity of CRISPR type IE in EC1390 was not further characterized.

### Conjugative plasmids pEC1390-1 and pEC1390-2

The Nanopore WGS results showed that two plasmids with replicons IncFIA (pEC1390-1) and IncFII (pEC1390-2) were found in EC1390 (Table 1). Moreover, conjugation-related genes were identified in pEC1390-1 and pEC1390-2. Plasmid pEC1390-1 contained a class 1 integron accumulated various antimicrobial resistance genes (ARGs), including *aadA5, aac(6’)-Ib-cr*, *bla*_CTX-M-15_, *bla*_OXA-1_, *bla*_NDM-5_, *catB3*, *catA1*, *sul1*, *dfrA17*, *tetA*, and *mph(A)*, and therefore conferred resistance to streptomycin, fluoroquinolone, aminoglycosides, β-lactams, chloramphenicol, sulfamethoxazole, trimethoprim aminoglycosides, tetracycline, trimethoprim, and azithromycin (Table 2), consistent with our antimicrobial susceptibility results. pEC1390-2 contained ARGs, *rmtB*, and *bla*_TEM-1B_, which conferred resistance to aminoglycoside and β-lactams, respectively (Table 2).

**Table 2:** Antimicrobial resistance genes are present in the pEC1390-1 and pEC1390-2

Plasmid	Antimicrobial resistance genes	Position in plasmid	Identity	Characteristics
**pEC1390-1**	*dfrA17*	4344.4812	98.95	trimethoprim-resistant dihydrofolate reductase
	*aadA5*	4942.5728	99.75	AadA family aminoglycoside 3″-O-nucleotidyltransferase
	*sul1*	6273.7110	99.76	sulfonamide-resistant dihydropteroate synthase
	*ble*^a^	11,043.11381	-	bleomycin binding protein Ble-MBL
	*bla*_NDM-5_	11,408.12218	99.75	NDM family subclass B1 metallo-β-lactamase
	*mph(A)*	13,597.14497	99.78	Mph(A) family macrolide 2’-phosphotransferase
	*mrx(A)*^a^	14,506.15734	-	macrolide resistance MFS transporter Mrx(A)
	*bla*_CTX-M-15_	20,707.21579	99.66	CTX-M family class A extended-spectrum β-lactamase
	*catB3*	22,940.23378	99.10	CatB-related O-acetyltransferase
	*bla*_OXA-1_	23,512.24338	99.52	OXA-1 family class D β-lactamase
	*aac(6')-Ib-cr*	24,467.24985	99.61	fluoroquinolone-acetylating aminoglycoside 6’-N-acetyltransferase AAC(6')-Ib-cr5
	*tet(A)*	28,507..29704	99.67	tetracycline efflux MFS transporter Tet(A)
	*K7G95_00640*^a^	29,736.30619	-	EamA family transporter
	*catA1*	154,061.154715	99.10	chloramphenicol acetyltransferase
**pEC1390-2**	*bla*_TEM-1B_	60,372..61228	95.54	TEM family class A β-lactamase
	*rmtB*	61,397.62139	98.28	amikacin, gentamicin, tobramycin, kanamycin resistance

^a^Antimicrobial resistance genes were identified only by using NCBI prokaryotic annotation pipeline but not ResFinder database

**Fig. 3:**
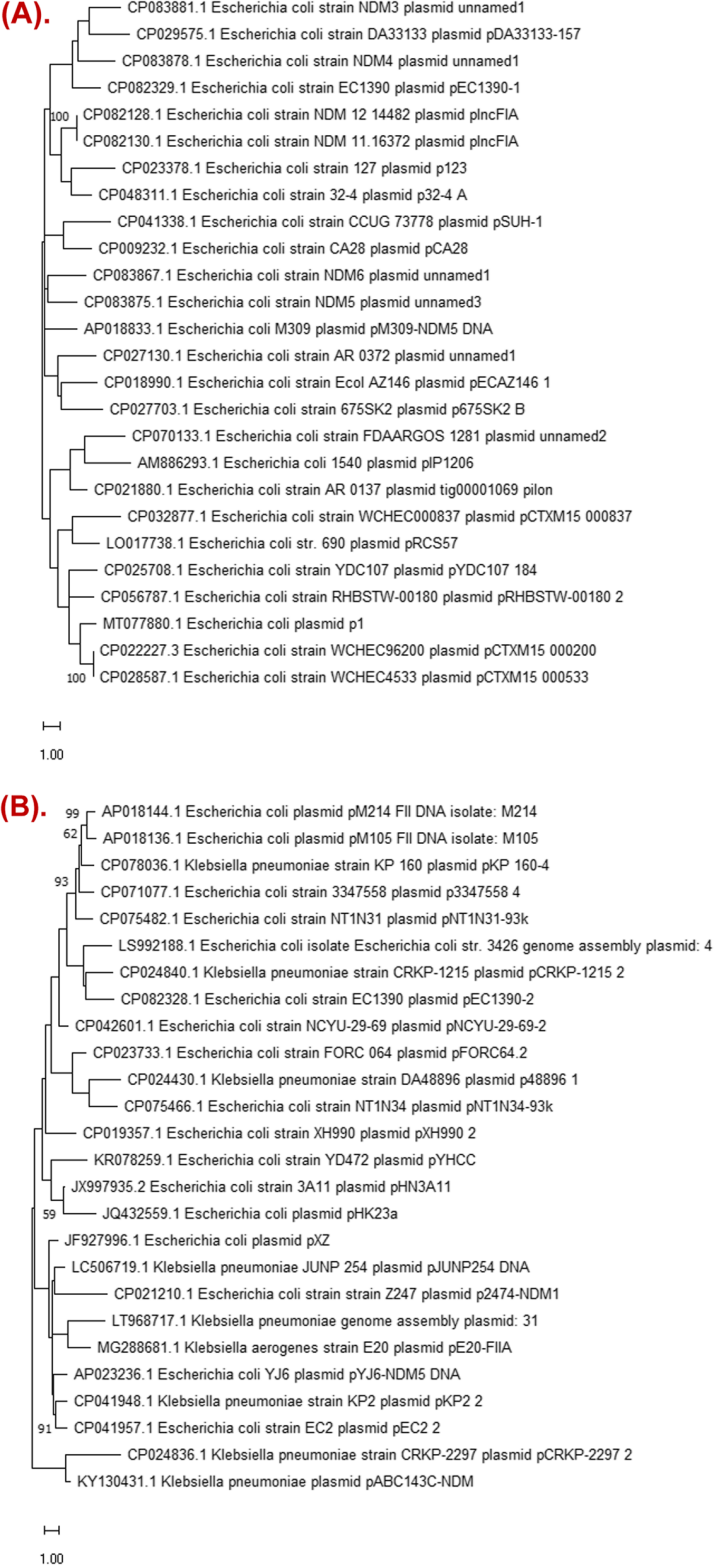
Phylogenetic tree of the plasmid close related to pEC1390-1 (A) and pEC1390-2 (B). The phylogenetic tree was based on the aligned nucleotide sequences of plasmids by using the Maximum Likelihood method. The percentage of trees in which the associated taxa clustered together is shown next to the branches. The tree is drawn to scale, with branch lengths measured in the number of substitutions per site. Bootstrap percentages ≥ 60% are shown at the nodes

Plasmid BLAST results showed that the sequence of pEC1390-1 was highly similar to plasmids *E. coli* strain NDM3 plasmid unnamed1 (accession number CP083881.1, coverage 88%, identity 99.27%), *E. coli* strain NDM4 plasmid unnamed1 (accession number CP083878.1, coverage 88%, identity 99.26%), NDM6 plasmid unnamed1 (accession number CP083867.1, coverage 88%, identity 99.26%), NDM5 plasmid unnamed3 (accession number CP083875.1, coverage 88%, identity 99.24%), and pIP1206 (accession number AM886293.1, coverage 82%, identity 98.93%). However, the *E. coli* strains NDM3, NDM4, NDM5, and NDM-6 belonged to the same clone. pIP1206 was identified in France. The phylogenetic tree indicated that pEC1390-1 showed the highest similarity to plasmid unnamed1 in *E. coli* strain NDM4, which was identified in *E. coli* containing *bla*_NDM-5_ in Austria, followed by NDM3 plasmid unnamed1 and pDA33133-157 (Fig. 3A). Interestingly, pDA33133-157 was identified in Sweden. These results suggest the distribution of pEC1390-1 similar *bla*_NDM_-positive plasmids worldwide.

The sequence of pEC1390-2 was highly similar to plasmids pNCYU-29–69-2 (accession number CP042601.1, coverage 100%, identity 99.16%), pHN3A11 (accession number JX997935.2, coverage 97%, identity 99.22%), pYHCC (accession number KR078259.1,coverage 95%, identity 99.31%), plasmid 31 (accession number LT968717.1.1, coverage 93%, identity 99.30%), and pKP_160-4 (accession number CP078036.1, coverage 92%, identity 99.30%). Interestingly, pNCYU-29–69-2 was identified in a *mcr*-3 positive *E. coli* from diseased swine. The phylogenetic tree showed that pEC1390-2 showed the highest similarity to plasmid pCRKP-1215_2 (coverage 92%, identity 99.02%) (Fig. 3B), which was identified in *Enterobacteriaceae* in South Korea.

### The biological function of plasmids pEC1390-1 and pEC1390-2

Compare to phylogenetic group B2, group A strains usually have fewer virulence factors and are less virulent [24]. Our WGS results showed that pEC1390-1 contained genes not only involved in antibiotic resistance but also iron acquisition and metabolism, protein metabolism, regulation and cell signaling, amino acid synthesis, and sulfur metabolism (Fig. S2). Therefore, we first performed conjugation to test the transferability of pEC1390-1 and pEC1390-2 (Fig. S3). The random amplified polymorphic DNA (RAPD)-PCR patterns of all TCGs were identical to the *E. coli* recipient C600 (Fig. S3A), and the *bla*_NDM_ was detected in EC1390, EC1390-TCG(L) (C600 contained pEC1390-1), and EC1390-TCG(S + L) (C600 contained pEC1390-1 and pEC1390-2) (Fig. S3B). Moreover, plasmid patterns determined by Kado-Liu plasmid extraction methods demonstrated that EC1390-TCG(L) contained pEC1390-1, EC1390-TCG(S) contained pEC1390-2, and EC1390-TCG(S + L) contained pEC1390-1 and pEC1390-2) (Fig. S3C). mCIM and eCIM were performed on EC1390 and TCGs to confirm the presence of NDM carbapenemase (Fig. S4). The results showed that EC1390, EC1390-TCG(L), and EC1390-TCG(L + S) were carbapenemase producers (Fig. S4). Moreover, recipient *E. coli* C600 was susceptible to all 21 antibiotics tested (Table S2). Compared to C600, EC1390-TCG(S) showed resistance to AN, AMC, AM, and GM, and EC1390-TCG(L) showed resistance to AMC, AM, CRO, CXM, ETP, IPM, TZP, SXT, SAM, FEP, FOX, CAZ, and TE (Table S2).

**Fig. 4:**
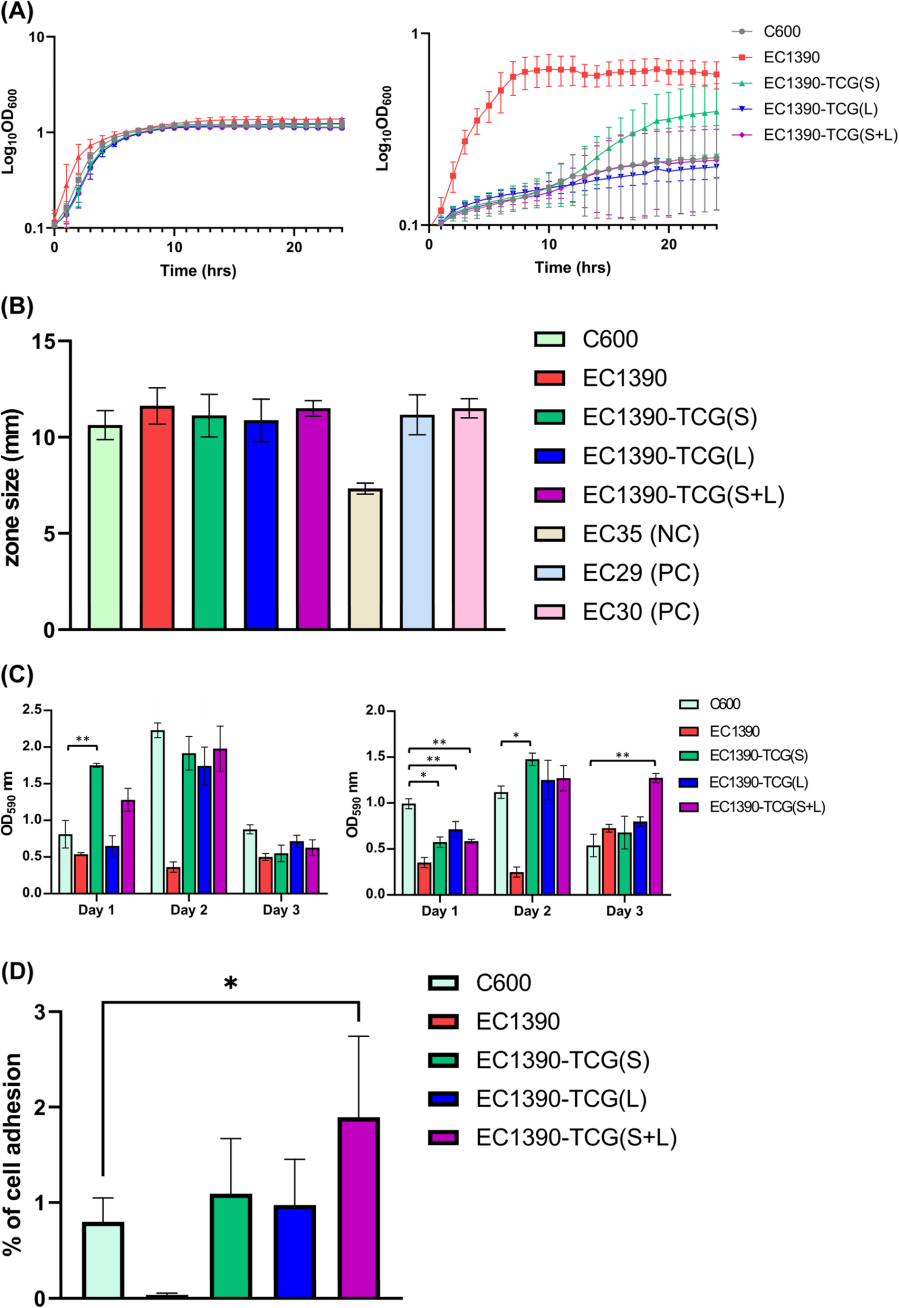
Characterization of phenotypes of EC1390, C600, and 3 transconjugants derevied from C600. **A** Growth curves of *E. coli* strains in LB (left panel) and M9 broth (right panel). **B** Siderophore activity was determined by CAS agar diffusion assay. The halo diameter represents siderophore activity was measured after 16 h incubation. **C** Biofilms of *E. coli* strains in LB (left panel) and M9 broth (right panel) were stained with crystal violet and measured at wavelength of 590 nm. **D** Bacterial adhesion assays in bladder epithelium cell line 5637. All experiments were performed in biological triplicate. Error bars represent the standard deviations of three independent experiments. The statistical analysis was performed using Student’s *t* test and the *p* values were provided (**, *p* < 0.01; *, *p* < 0.05). PC, positive control; NC, negative control

To characterize the biological function of two plasmids in EC1390 in addition to antibiotic resistance, we compared the bacterial growth rate, biofilm formation, iron acquisition and bladder epithelial cell adhesion, between EC1390, C600, and 3 TCGs (Fig. 4). Growth curves of *E. coli* strains were measured in triplicate for each of M9 broth (nutrition-limited) and LB broth (Fig. 4A). There was no significant difference in the growth rates between 5 strains in LB. Compared to C600 and 3 TCGs, UPEC 1390 showed a higher growth rate in M9 broth. Interestingly, EC1390-TCG(S) showed a significant increase in growth rate in M9 broth (*p* < 0.05), compared to C600, EC1390-TCG(S), and EC1390-TCG(S + L) (Fig. 4A). Our results indicated that 5 examined strains showed a similar ability of iron acquisition (Fig. 4B). The results of biofilm formation in LB showed that EC1390-TCG(S) had an increase in biofilm formation after 1-day incubation (Fig. 4C). All 3 TCGs showed lower biofilm formation in M9 broth after 1-day incubation, compared to the C600, but EC1390-TCG(S) and EC1390-TCG(S + L) had increasing biofilm formation in M9 after 2-day and 3-day incubation, respectively (Fig. 4C). Moreover, EC1390-TCG(S + L) had a higher cell adhesion ability compared to the C600 (Fig. 4D).

## Discussion

In this study, we combined Nanopore WGS with phenotypic assays to characterize an NDM-5-producing *E. coli* isolated from UTI patient. EC1390 was isolated from the urine sample on day 14 of patient’s hospitalization. Cefepime, doxycycline, and sulfamethoxazole/trimethoprim, were used for the treatment of bacterial infections before EC1390 was isolated (Fig. 1). The history of antibiotic therapies has been reported to be associated with the development of antibiotic resistance [25], however, the limit of our study is that we did not isolate EC1390 before antibiotic therapies to evaluate the association between the therapy and resistance development. On day 18 of the hospitalization, a carbapenem-resistant *E. coli* (CREC) was isolated from the patient’s rectal swab, however, we did not further determine the clonality of CREC isolated from urine and rectal swab.

The inconsistency of annotation results from NCBI PGAP and RAST server suggests the assemblies generated only from MinION data contained many indels and substitutions that affected the following analyses (Table 1 and Fig. S2). In addition, in the presence of a frameshift, the NCBI PGAP annotation pipeline generates a ‘corrected’ sequence of the genome, adding or subtracting bases to repair the translation frame [26]. Therefore, the number of coding sequences predicted by NCBI PGAP annotation was lower than the RAST server. Moreover, our Kado-Liu results showed the other 3 plasmids with sizes below 10 kbp, however, Nanopore WGS results showed only two large plasmids (Fig. 2 and Fig. S1). These results suggest the loss of small plasmids by using long-read Nanopore WGS platform. Many studies showed that the Nanopore/Illumina hybrid sequencing platform enhances the accuracy of genome assemblies [27]. However, this study aimed to combine WGS and phenotypic assays to characterize EC1390, therefore, we did not use a hybrid sequencing platform to further verify the EC1390 genome. Instead, we increased the depth of coverage to enhance the accuracy of EC1390 genome.

Our results showed that EC1390 belonged to the O101-H10 serotype, phylogenetic group A, and untypable sequence type *E. coli*. The phylogenetic groups A, B1, and D had fewer virulence determinants than B2 strains, and O101-H10 serotype phylogenetic group A *E. coli* was relatively rare reported [24, 28]. Mamani et al. reported that of the 2,427 *E. coli* bloodstream isolates in a Spanish hospital, 96 (4.0%) were extended-spectrum β-lactamase producers. Moreover, only 2 out of 96 ESBL-producer belonged to the O101-H10 serotype (1 ST32 strain contained *bla*_CTX-M-32_, and 1 ST617 strain contained *bla*_CTX-M-14_) [28]. Therefore, the characteristics of O101-H10 serotype pathogenic *E. coli* were rarely determined. Currently, XDR*-E. coli* ST7624 belonged to O − :H34 serotype harboring *bla*_CTX−M−14b_ was identified from milk samples [29]. The characteristics and circulation of these XDR pathogenic *E. coli* that can cause human diseases should be monitored.

Currently, international circulation of NDM-5-producing *E. coli* was reported but only a few UPEC isolates harboring *bla*_NDM-5_ were identified [30–32]. To our knowledge, there are only 2 studies that reported the *E. coli* carrying *bla*_NDM-5_ in Taiwan [33, 34]. Chang et al. collected 237 carbapenem-nonsusceptible *E. coli* from 2012 to 2015, and only one ST117 *E. coli* contained *bla*_NDM-5_ [34]. From 2016 to 2018, Huang et al. showed 78 (0.5%) of the 17,018 *E. coli* clinical isolates collected at National Taiwan University Hospital were carbapenem-non-susceptible. Moreover, among the 78 carbapenem-nonsusceptible *E. coli* isolates, 23 (29.5%) were carbapenemase-producing *E. coli*, and 5 NDM-5-producers were identified (ST38, 167, 359, 410 (*n* = 2)) [33]. However, our results showed the distribution of pEC1390-1 similar *bla*_NDM-5_-positive plasmids worldwide. Therefore, it is worth monitoring the spread of *bla*_NDM-5_-positive plasmids in Taiwan.

WGS is becoming widely used in pathogen identification, genome characterization, and phenotypic prediction. However, the virulence of plasmids carrying *bla*_NDM-5_ is less investigated. Although our results showed that the plasmid pEC1390-2 promoted the growth of bacteria in nutrition-limited M9 broth (Fig. 4A), however, we could not find out the potential annotated genes involved in this phenotypic change. There are 17 hypothetical proteins identified on pEC1390-2. Therefore, the roles of these genes in bacterial growth remain to be identified. Although our results indicated that pEC1390-1 and pEC1390-2 were not involved in iron acquisition (Fig. 4B), we still can not rule out the possibility that these two plasmids may play critical roles in biofilm formation and iron acquisition in vivo. Moreover, our recipient C600 showed high biofilm formation and iron acquisition, even compared to EC1390, a UPEC strain. These results showed that C600 may not a suitable for these two phenotypic assays.

Our results showed that the biofilm formation of TCGs compared to C600 was significantly different in different broth and incubation periods (Fig. 4C). Moreover, EC1390-TCG(S + L) had a higher cell adhesion ability compared to the C600 (Fig. 4D). These results suggest the cross-talk between pEC1390-1, pEC1390-2, and chromosomal genes, in different culture conditions and growth stages. Therefore, a transcriptome analysis to determine the cross-talk between genes located on chromosomal and plasmids would be useful to further elucidate the function(s) of two plasmids in UPEC.

## Conclusions

In summary, we characterized an NDM-5-producing *E. coli* isolated from UTI patients by using a combination of WGS and phenotypic assays. We also demonstrated that these two conjugative plasmids were involved in antibiotic resistance, bacterial growth, biofilm formation, and bacterial adhesion to host cells.

## Supplementary Information

**Additional file 1:** **Fig. S1. **Profiles of plasmids of *E. coli *EC1390**. ***E. coli* DH5α was used as a negative control. *Salmonella* OU7526 and *E. coli* EC974 contained 2 (50 and 90 kbp) and 3 (78, 92, and 105 kbp) plasmids, respectively, were used as plasmid size controls. GeneRuler 1 kb DNA ladder was used as a size marker. The experiment was conducted in duplicate.**Fig. S2.** (A). Subsystem distribution of *E. coli* EC1390 chromosome based on the RAST annotation server. Out of 10,738 coding sequences predicted by RAST server, the subsystem coverage is 64% which contributes to a total of 599 subsystems. (B). Subsystem distribution of *E. coli* pEC1390-1 plasmid based on RAST annotation server. Out of 395 coding sequences predicted by RAST server, the subsystem coverage is 33% which contributes to a total of 13 subsystems. The green bar of the subsystem coverage indicates the percentage of the proteins included in the subsystems while the blue bar refers to the percentage of the proteins that are not included in the subsystems. **Fig. S3.** Verification of EC1390 transconjugants. (A). Random amplified polymorphic DNA (RAPD) patterns of *E. coli *recipient C600, EC974, EC1515, and TCGs. EC974 was used as a conjugation positive control. (B). PCR analysis to detect the *bla*_NDM-5_ gene. (C). Plasmid profiles of *E. coli *recipient and transconjugants. *E. coli* C600 was used as a negative control. *Salmonella* OU7526, *E. coli* EC974, and EC1515, contained 2 (50 and 90 kbp), 3 (78, 92, and 105 kbp), and 3 (78, 92, and 105 kbp) plasmids, respectively, were used as plasmid size controls. The experiment was conducted in duplicate. NC, negative control; TGC, transconjugant; Marker, 100-bp DNA ladder. **Fig. S4.** mCIM and eCIM tests of EC1390 and its derived transconjugants. The inhibition zone diameter (mm) is shown in parentheses. According to the CLSI guidelines, *K. pneumoniae* ATCC BAA-1706 (carbapenemase negative), *K. pneumoniae* ATCC BAA-1705 (*bla*_KPC_ positive), and *K. pneumoniae* ATCC BAA-2146 (*bla*_NDM_ positive) were used as internal controls for mCIM and eCIM tests. The mCIM and eCIM tests were replicated by two independent investigators to ensure reproducibility. **Table S1.** EC1390 CRISPR type IE spacer sequences and their plasmid and/or phage targets. **Table S2.** Antimicrobial susceptibility of *E. coli* recipient and transconjugants. 

## Data Availability

Whole genome sequencing data are available in the NCBI database under BioProject accession PRJNA758726. The complete genome sequences of the *E. coli* strain EC1390 have been deposited in GenBank under the accession numbers CP082330 (EC1390 chromosome), CP082329 (pEC1390-1), and CP082328 (pEC1390-2).

## Abbreviations

ARG: Antimicrobial resistance genes
CREC: Carbapenem-resistant *E. coli*
NCKUH: National Cheng-Kung University Hospital
NGS: Next-generation sequencing
RAPD: Random amplified polymorphic DNA
TCG: Transconjugants
UPEC: Uropathogenic *E. coli*
UTI: Urinary tract infection
WGS: Whole-genome sequencing
XDR: Extensively-drug resistant
