# Supplementary material for: Whole-genome-sequence-based characterization of an NDM-5-producing uropathogenic Escherichia coli EC1390
Source: BMC Microbiol. 2022 Jun 6;22:150. doi: 10.1186/s12866-022-02562-6 (PMC9172118; doi:10.1186/s12866-022-02562-6)
Supplement: Supplementary file 1 — Additional file 1: Fig. S1. Profiles of plasmids of E. coli EC1390. E. coli DH5α was used as a negative control. Salmonella OU7526 and E. coli EC974 contained 2 (50 and 90 kbp) and 3 (78, 92, and 105 kbp) plasmids, respectively, were used as plasmid size controls. GeneRuler 1 kb DNA ladder was used as a size marker. The experiment was conducted in duplicate.Fig. S2. (A). Subsystem distribution of E. coli EC1390 chromosome based on the RAST annotation server. Out of 10,738 coding sequences predicted by RAST server, the subsystem coverage is 64% which contributes to a total of 599 subsystems. (B). Subsystem distribution of E. coli pEC1390-1 plasmid based on RAST annotation server. Out of 395 coding sequences predicted by RAST server, the subsystem coverage is 33% which contributes to a total of 13 subsystems. The green bar of the subsystem coverage indicates the percentage of the proteins included in the subsystems while the blue bar refers to the percentage of the proteins that are not included in the subsystems. Fig. S3. Verification of EC1390 transconjugants. (A). Random amplified polymorphic DNA (RAPD) patterns of E. coli recipient C600, EC974, EC1515, and TCGs. EC974 was used as a conjugation positive control. (B). PCR analysis to detect the blaNDM-5 gene. (C). Plasmid profiles of E. coli recipient and transconjugants. E. coli C600 was used as a negative control. Salmonella OU7526, E. coli EC974, and EC1515, contained 2 (50 and 90 kbp), 3 (78, 92, and 105 kbp), and 3 (78, 92, and 105 kbp) plasmids, respectively, were used as plasmid size controls. The experiment was conducted in duplicate. NC, negative control; TGC, transconjugant; Marker, 100-bp DNA ladder. Fig. S4. mCIM and eCIM tests of EC1390 and its derived transconjugants. The inhibition zone diameter (mm) is shown in parentheses. According to the CLSI guidelines, K. pneumoniae ATCC BAA-1706 (carbapenemase negative), K. pneumoniae ATCC BAA-1705 (blaKPC positive), and K. pneumoniae ATCC BAA-2146 (blaNDM posi [file 12866_2022_2562_MOESM1_ESM.doc]

**Fig. S1**


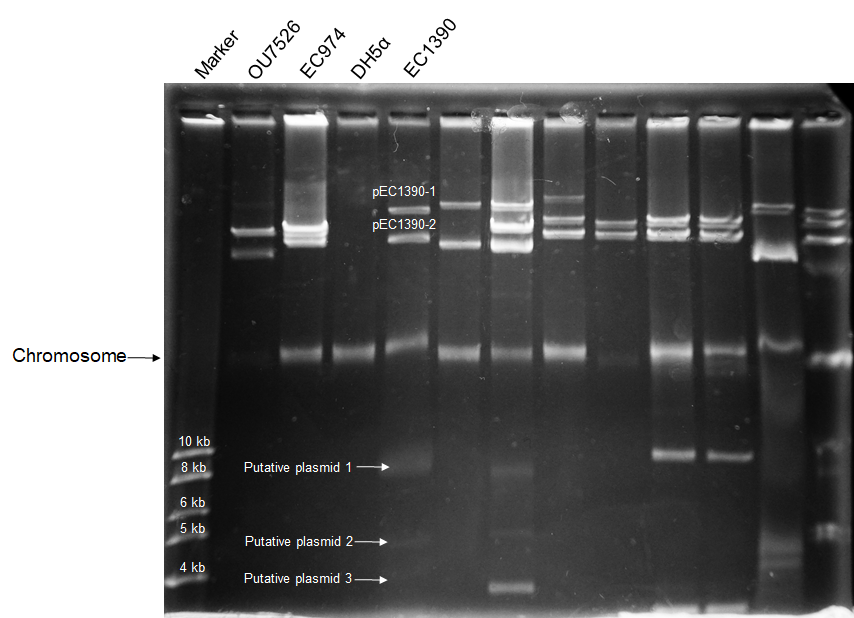


**Fig. S1. Profiles of plasmids of *E. coli* EC1390.** *E. coli* DH5α was used as a negative control. *Salmonella* OU7526 and *E. coli* EC974 contained 2 (50 and 90 kbp) and 3 (78, 92, and 105 kbp) plasmids, respectively, were used as plasmid size controls. GeneRuler 1 kb DNA ladder was used as a size marker. The experiment was conducted in duplicate.

**Fig. S2**

**(A).**


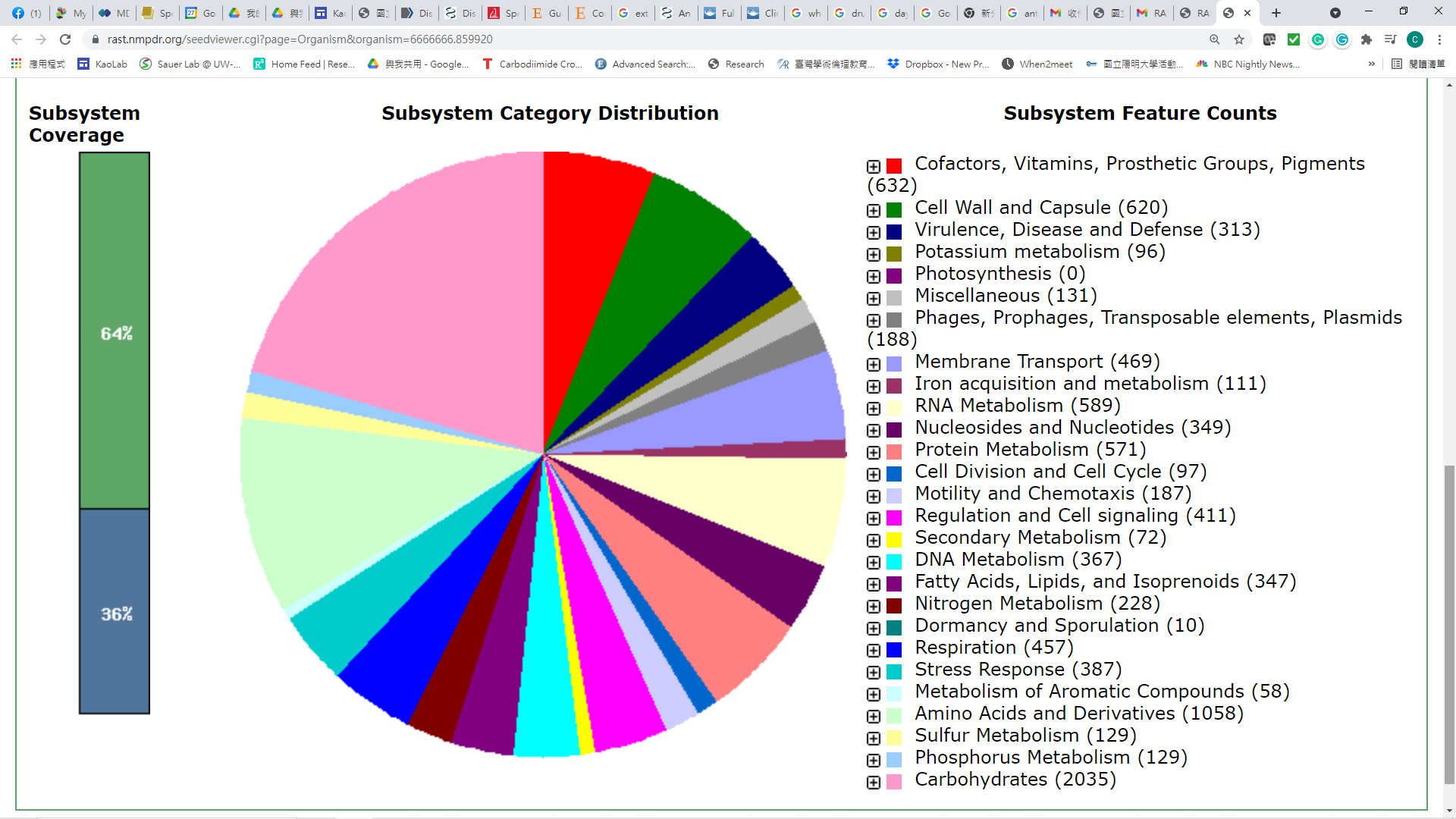


**(B).**


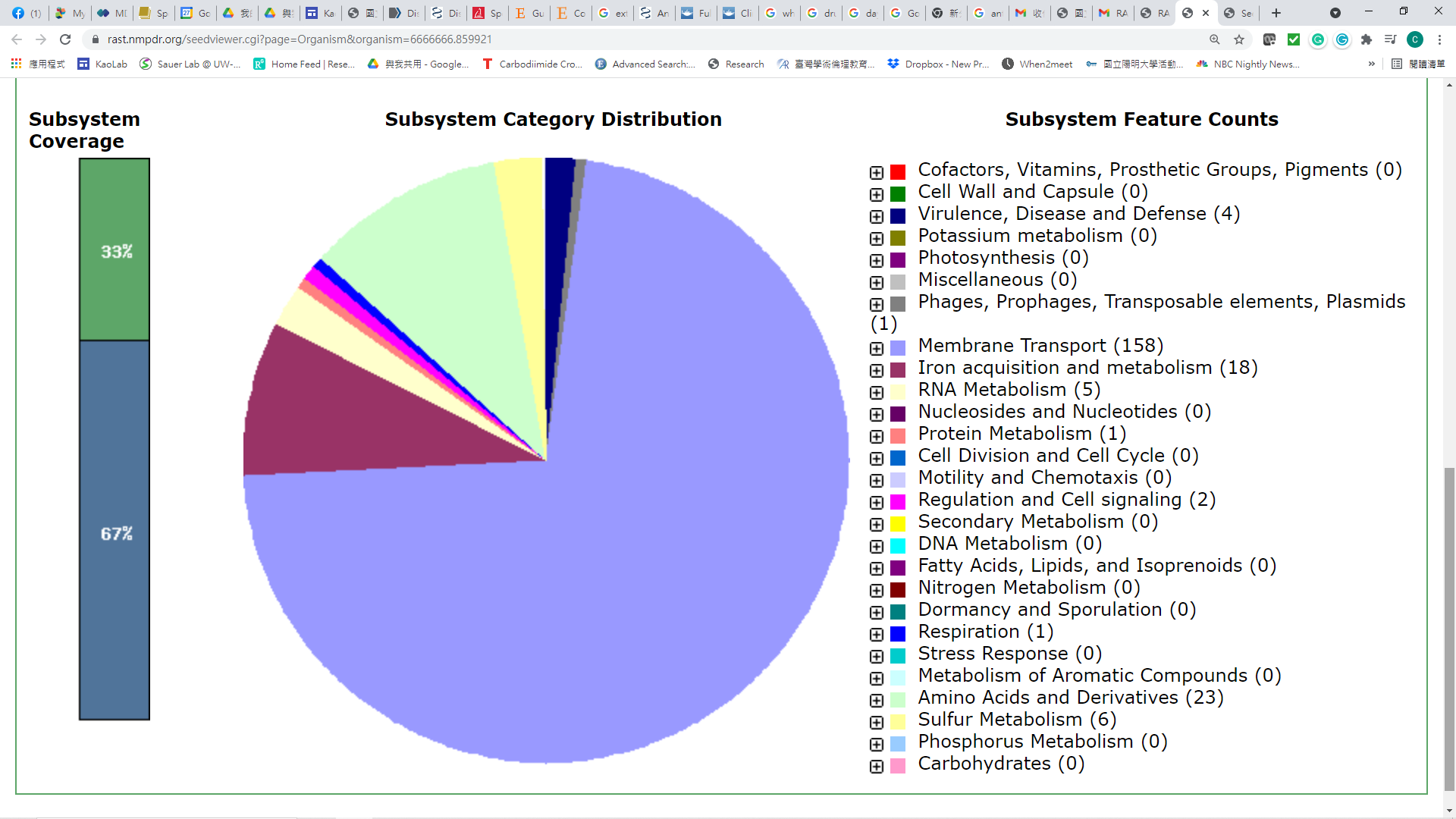


**Fig. S2.** (A). Subsystem distribution of E. coli EC1390 chromosome based on the RAST annotation server. Out of 10,738 coding sequences predicted by RAST server, the subsystem coverage is 64% which contributes to a total of 599 subsystems. (B). Subsystem distribution of E. coli pEC1390-1 plasmid based on RAST annotation server. Out of 395 coding sequences predicted by RAST server, the subsystem coverage is 33% which contributes to a total of 13 subsystems. The green bar of the subsystem coverage indicates the percentage of the proteins included in the subsystems while the blue bar refers to the percentage of the proteins that are not included in the subsystems.

**Fig. S3**

**(A).**


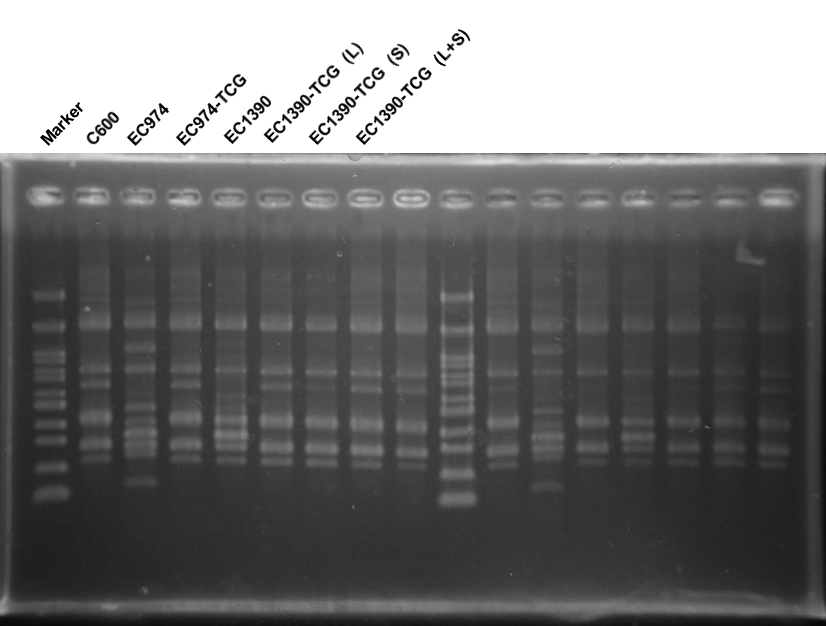


**(B).**


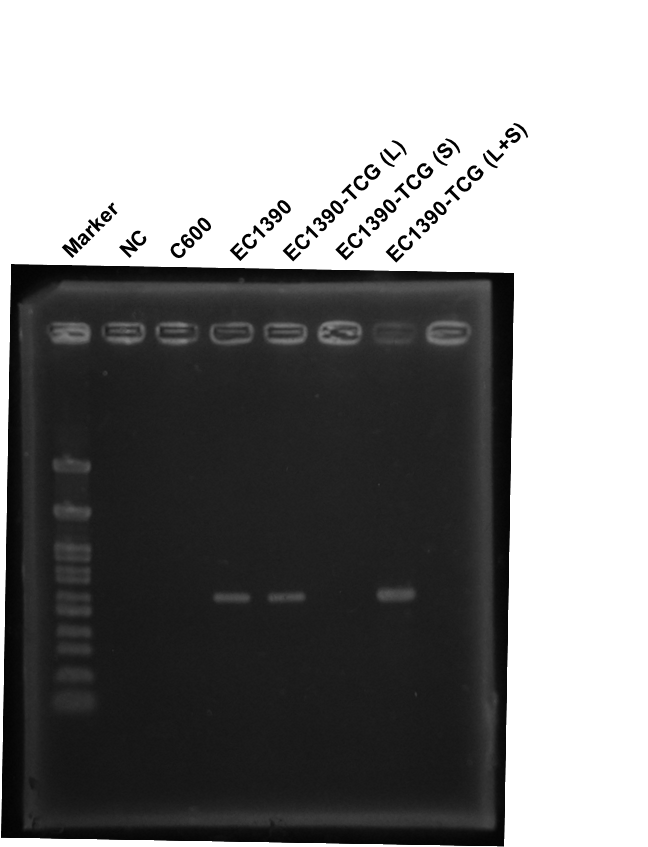


**(C).**


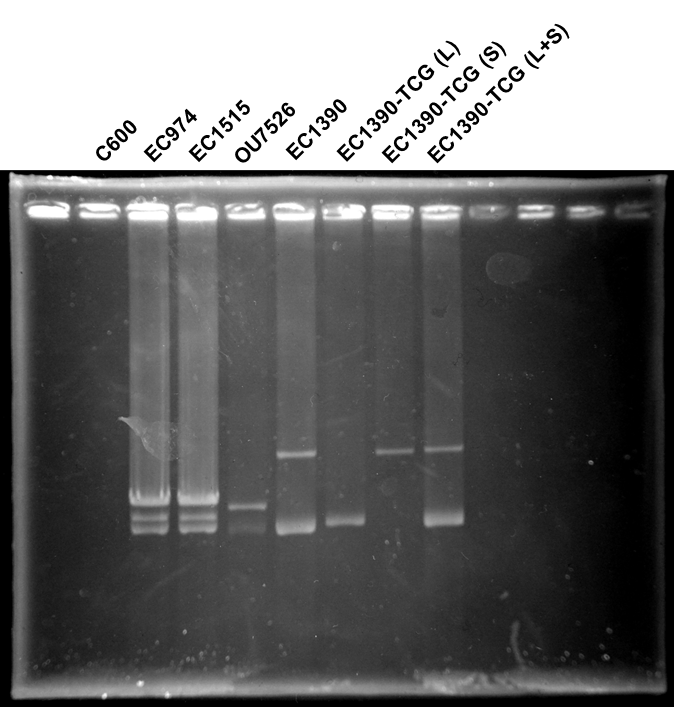


**Fig. S3.** Verification of EC1390 transconjugants. (A). Random amplified polymorphic DNA (RAPD) patterns of *E. coli* recipient C600, EC974, EC1515, and TCGs. EC974 was used as a conjugation positive control. (B). PCR analysis to detect the *bla*_NDM-5_ gene. (C). Plasmid profiles of *E. coli* recipient and transconjugants. *E. coli* C600 was used as a negative control. *Salmonella* OU7526, *E. coli* EC974, and EC1515, contained 2 (50 and 90 kbp), 3 (78, 92, and 105 kbp), and 3 (78, 92, and 105 kbp) plasmids, respectively, were used as plasmid size controls. The experiment was conducted in duplicate. NC, negative control; TGC, transconjugant; Marker, 100-bp DNA ladder.

**Fig. S4**


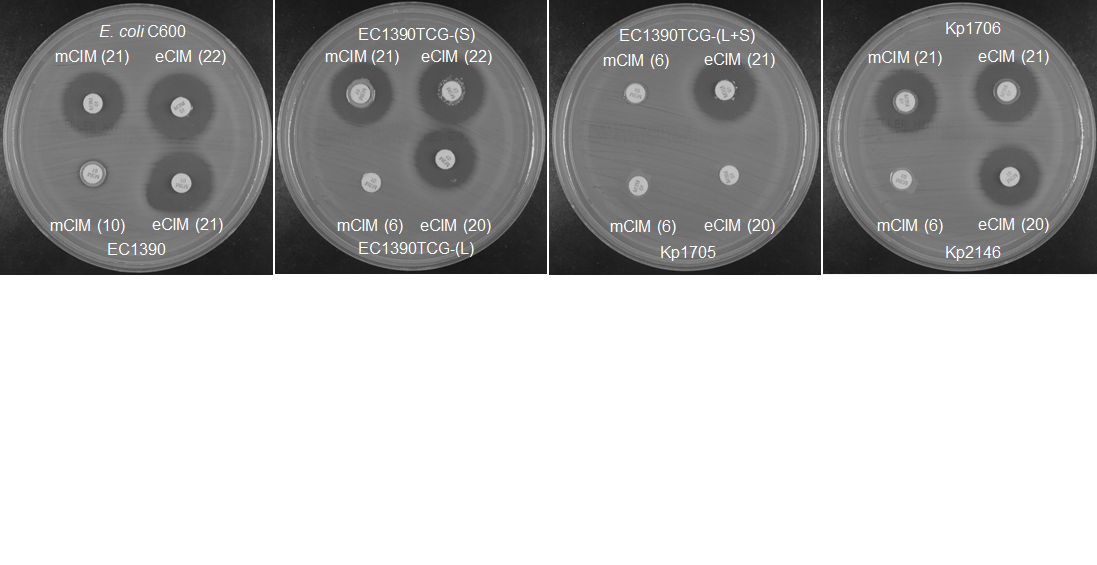
 Fig. S4. mCIM and eCIM tests of EC1390 and its derived transconjugants. The inhibition zone diameter (mm) is shown in parentheses. According to the CLSI guidelines, K. pneumoniae ATCC BAA-1706 (carbapenemase negative), K. pneumoniae ATCC BAA-1705 (bla_KPC_ positive), and K. pneumoniae ATCC BAA-2146 (bla_NDM_ positive) were used as internal controls for mCIM and eCIM tests. The mCIM and eCIM tests were replicated by two independent investigators to ensure reproducibility.

**Table S1. EC1390 CRISPR type IE spacer sequences and their plasmid and/or phage targets.**

| **Spacer** | **Sequences** | **Plasmid and/or phage targets** |
| --- | --- | --- |
| 1 | TGTGTTTGCGGCATTAACGCTCACCAGTATTTC | PHAGE_Entero_mEp235PHAGE_Entero_mEp390*Klebsiella aerogenes* strain NCTC9644 plasmid 5*Klebsiella pneumoniae* strain NCTC9793 plasmid 6PHAGE_Entero_SfV |
| 2 | CGACGTGGTCATGGGTGCTGCTGTTGCAGAGCCA | 1. *Arthrobacter* sp. strain ANT_H40 plasmid pA40H1 2. *Streptomyces fungicidicus* strain TXX3120 plasmid p1 |
| 3 | GAGCAGATACACGGCTTTGTATTCCGTGCGCCC | - |
| 4 | GAATAGCAATAGTCCATAGATTTGCGAAAACAGG | - |
| 5 | GGAGCCTGACGAGACTACTGAGGCCGTTCTGTC | - |
| 6 | TTTGGATCGGGTCTGGAATTTCTGAGCGGTCGC | - |
| 7 | CGAATCGCGCATACCCTGCGCGTCGCCGCCTGC | PHAGE_Strept_VWB |
| 8 | TCAGCTTTATAAATCCGGAGATACGGAAACTA | - |
| 9 | AGACTCACCCCGAAAGAGATTGCCAGCCAGCTTG | - |
| 10 | CTGCTGGAGCTGGCTGCAAGGCAAGCCGCCCAG | - |

**Table S2. Antimicrobial susceptibility of *E. coli*** recipient and transconjugants.

|  | **Antimicrobial agents^a^** | | | | | | | | | | | | | | | | | | | | |
| --- | --- | --- | --- | --- | --- | --- | --- | --- | --- | --- | --- | --- | --- | --- | --- | --- | --- | --- | --- | --- | --- |
| Strains | AN | AMC | AM | CMZ | CRO | CXM | CIP | ETP | GM | IPM | MEM | LVX | TZP | SXT | SAM | FEP | FOX | CAZ | TGC | TE | CL |
| EC1390 | R  (≥64) | R (>32/16) | R (>32) | R  (≥64) | R  (≥64) | R  (≥64) | R  (≥4) | R  (≥8) | R  (≥16) | R  (8) | R  (≥16) | R  (≥8) | R  (≥128) | R  (≥320) | R (>32) | R (>64) | R (>64) | R (>64) | S  (≤0.5) | R  (≥16) | S  (≤0.5) |
| C600 | S (≤2) | S (≤8/  4) | S  (8) | S (≤1) | S (≤1) | S  (4) | S (≤0.25) | S (≤0.5) | S  (≤1) | S (≤0.25) | S (≤0.25) | S (≤0.12) | S  (≤4) | S (≤20) | S (≤2) | S (≤1) | S (≤4) | S (≤1) | S  (≤0.5) | S  (2) | S  (≤0.5) |
| EC1390-TCG(S) | R  (≥64) | R (>32/16) | R (>32) | S (≤1) | S (≤1) | S  (4) | S (≤0.25) | S (≤0.5) | R  (≥16) | S (≤0.25) | S (≤0.25) | S (≤0.12) | S  (≤4) | S (≤20) | S  (8) | S (≤1) | S (≤4) | S (≤1) | S  (≤0.5) | S  (2) | S  (≤0.5) |
| EC1390-TCG(L) | S (≤2) | R (>32/16) | R (>32) | S (≤4) | R  (≥64) | R  (≥64) | S (≤0.25) | R  (2) | S  (≤1) | R (8) | S  (1) | S (≤0.12) | R  (≥128) | R  (≥320) | R (>32) | R  (2) | R (>64) | R (>64) | S  (≤0.5) | R  (≥16) | S  (≤0.5) |
| EC1390-TCG(S+L) | R  (≥64) | R (>32/16) | R (>32) | R  (≥64) | R  (≥64) | R  (≥64) | R  (≥4) | R  (4) | R  (≥16) | R  (≥16) | R  (≥16) | R  (≥8) | R  (≥128) | R  (≥320) | R (>32) | R (>64) | R (>64) | R (>64) | S  (≤0.5) | R  (≥16) | S  (≤0.5) |

**^a^** The minimal inhibitory concentration to antibiotics (μg/mL) determined by Vitck 2 system is shown in parentheses.

EC1390-TCG(S), C600 contained pEC1390-2; EC1390-TCG(L), C600 contained pEC1390-1; EC1390-TCG(S+L), C600 contained pEC1390-1 and pEC1390-2. AmC, amoxicillin with clavulanic acid; AN, amikacin; AM, ampicillin; CMZ, cefmetazole; CRO, ceftriaxone; CXM, cefuroxime; CIP, ciprofloxacin; ETP, ertapenem; GM, gentamicin; IPM, imipenem; MEM, meropenem; LVX, levofloxacin; TZP, piperacillin/tazobactam; SXT, sulfamethoxazole with trimethoprim; SAM, ampicillin with sulbactam; FEP, cefepime; FOX, cefixitin; CAZ, ceftazidime; TGC, tigecycline; TE, tetracycline; CL, colistin. R, resistant; S, susceptible.
